# A prognostic nomogram for recurrence survival in post-surgical patients with varicose veins of the lower extremities

**DOI:** 10.1038/s41598-024-55812-0

**Published:** 2024-03-06

**Authors:** Hai Hu, Lili Hu, Ziqing Deng, Qihua Jiang

**Affiliations:** 1 Department of General Surgery, The Third Hospital of Nanchang, No. 2, Xiangshan South Road, Xihu District, Nanchang, Jiangxi China; 2 Department of pediatrics, The Third Hospital of Nanchang, Nanchang, China; 3 Department of Breast Surgery, The Third Hospital of Nanchang, Nanchang, China

**Keywords:** Varicose veins of the lower extremities, Recurrence survival, Prognostic model, Nomogram, LASSO regression, Risk factors, Signs and symptoms

## Abstract

Varicose veins of the lower extremities (VVLEs) are prevalent globally. This study aims to identify prognostic factors and develop a prediction model for recurrence survival (RS) in VVLEs patients after surgery. A retrospective analysis of VVLEs patients from the Third Hospital of Nanchang was conducted between April 2017 and March 2022. A LASSO (Least Absolute Shrinkage and Selection Operator) regression model pinpointed significant recurrence predictors, culminating in a prognostic nomogram. The model’s performance was evaluated by C-index, receiver operating characteristic (ROC) curves, calibration plots, and decision curve analysis (DCA). The LASSO regression identified seven predictors for the nomogram predicting 1-, 2-, and 5-year RS. These predictors were age, body mass index (BMI), hypertension, diabetes, the Clinical Etiological Anatomical Pathophysiological (CEAP) grade, iliac vein compression syndrome (IVCS), and postoperative compression stocking duration (PCSD). The nomogram’s C-index was 0.716, with AUCs (Area Under the Curve scores) of 0.705, 0.725, and 0.758 for 1-, 2-, and 5-year RS, respectively. Calibration and decision curve analyses validated the model’s predictive accuracy and clinical utility. Kaplan–Meier analysis distinguished between low and high-risk groups with significant prognostic differences (*P* < 0.05). This study has successfully developed and validated a nomogram for predicting RS in patients with VVLEs after surgery, enhancing personalized care and informing clinical decision-making.

## Introduction

Varicose veins of the lower extremity (VVLEs) are a widespread clinical condition, with prevalence rates spanning from 14 to 64% across the general population. The more advanced stages of VVLEs impact an estimated 3.3% to 9.6% of individuals^1,2^. This condition is characterized by a range of symptoms, including leg pain, swelling, itching, fatigue, and, in more severe cases, changes in skin and soft tissue such as pigmentation, eczema, skin hardening, and ulceration. These symptoms can significantly diminish a patient’s quality of life^3,4^.

The spectrum of treatment options for VVLEs ranges from conservative measures, like medication and compression therapy, to surgical interventions, including high ligation, stripping, laser therapy, and radiofrequency ablation^5,6^. Although surgical treatments can alleviate symptoms and improve the quality of life, the recurrence rates post-operation are notably high, with estimates suggesting a 25% to 50% recurrence within five years^7,8^. Thus, accurately predicting a patient’s risk of post-surgical recurrence is crucial for tailoring personalized treatment plans and follow-up strategies.

Recent advancements have further enriched our understanding of VVLEs and their management. Notably, there has been a growing interest in minimally invasive techniques and their role in reducing recovery time, minimizing complications, and potentially lowering recurrence rates. Technologies such as endovenous laser ablation (EVLA) and radiofrequency ablation (RFA) have emerged as promising alternatives to traditional surgery, with recent studies highlighting their efficacy and patient satisfaction scores^9,10^. Moreover, the role of genetic predisposition and molecular biology in the development and progression of VVLEs is gaining attention^11^, suggesting potential areas for therapeutic intervention and risk assessment.

Previous studies have focused on factors influencing postoperative recurrence, previously identified as age, genetics, gender, body mass index (BMI), occupational habits (prolonged standing or sitting), and disease severity (CEAP grade)^12–14^. Limitations of earlier studies include small, single-center samples and basic statistical analyses, restricting the accuracy and applicability of their findings.

In response, we introduce the Nomogram model, a graphical predictive tool integrating multiple prognostic factors to yield a precise predictive score. Already successful in forecasting outcomes in cancers, cardiovascular, and chronic kidney diseases, this study explores the Nomogram model’s efficacy in predicting postoperative recurrence risk in VVLEs patients.

## Materials and methods

### Study population

**Figure 1:**
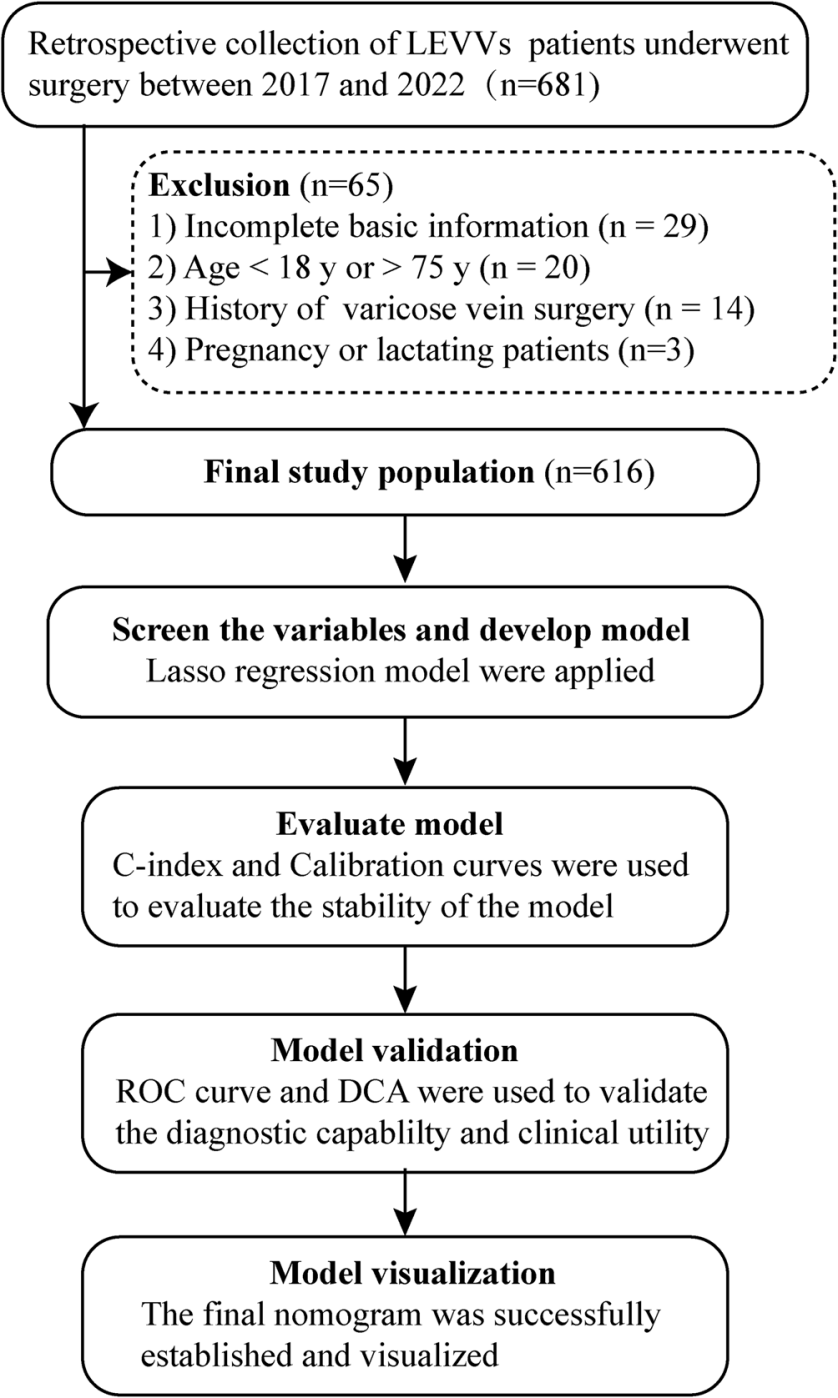
Flow diagram of study design. LEVVs, lower extremity varicose veins.

This study retrospectively analyzed patients with primary VVLEs admitted to the Third Hospital of Nanchang City between April 2017 and March 2022. Eligible participants were adults aged 18 to 75 years with Doppler ultrasound and/or venography-confirmed VVLEs, without concurrent lower limb injuries or fractures, and had at least one postoperative follow-up. Exclusions included incomplete demographic data, prior varicose vein surgery, or pregnancy/lactation. Recurrence post-surgery was defined as persistence or return of varicose veins^15,16^. Study flow is illustrated in Fig. 1.

### Data selection

Research data were collected on gender, age, Body Mass Index (BMI), smoking and drinking history, hypertension, diabetes, the Clinical Etiological Anatomical Pathophysiological (CEAP) grade, iliac vein compression syndrome (IVCS) diagnosis (confirmed by CT venography^17^), postoperative complications (PC), and postoperative compression stocking duration (PCSD).

For simplicity, BMI was categorized as < 25 kg/m^2^ or ≥ 25 kg/m^2^; CEAP grading as ≤ 4 or > 4; and compression stocking duration as < 3 months or ≥ 3 months^18,19^. Recorded complications included deep vein thrombosis, hemorrhage, infection, nerve damage, venous inflammation, skin pigmentation, varicose vein recurrence, and lymphedema.

### Follow-up

Follow-up was conducted until April 2023, with initial quarterly and then annual visits post-surgery. It included clinical evaluations and ultrasound scans to assess recovery and detect recurrence.

### Statistical analyses

The statistical analyses for this study were conducted using R software (version 4.1.2). The baseline characteristics between the recurrence and non-recurrence groups were compared using the Chi-square test and Fisher’s exact test. LASSO regression analysis was performed using the ‘glmnet’ package in R. By applying the ‘cox’ family, the Cox proportional hazards model was implemented in the LASSO analysis to select the most predictive variables for survival. Survival analysis was conducted and a nomogram was created using the ‘rms’ and ‘survival’ packages in R. packages in R. The ‘survminer’ package was used to visualize the survival analysis. The performance of the nomogram was assessed by generating a receiver operating characteristic (ROC) curve with the ‘timeROC’ and ‘pROC’ packages. The predictive accuracy and discriminative ability of the nomogram were further evaluated by Harrell’s concordance index (C-index) and the area under the curve (AUC)^20^. Calibration of the nomogram was performed by generating a calibration curve with the ‘calibrationCurves’ package to assess the agreement between the predicted and observed outcomes. We also conducted a decision curve analysis (DCA) using the ‘DCA.R’ or ‘rmda’ packages to evaluate the clinical usefulness and net benefit of the nomogram. X-tile software is used to find the best cutoff value for the total point, patients in both groups were then classified into high, medium and low risk groups. Survival was compared using Kaplan–Meier curves with log-rank tests. *P*-value < 0.05 (two-sided) was considered as statistically significant.

### Ethics statement

Ethical approval was obtained from the hospital’s ethics committee, adhering to the Helsinki Declaration. Written informed consent was obtained from all participants, in accordance with the Helsinki Declaration.

## Results

### Patient characteristics

In this study, 616 patients met the inclusion criteria. They were followed for a median duration of 16.8 months post-VVLEs surgery. Recurrence was observed in 84 (13.6%) patients. Based on recurrence, participants were divided into two cohorts: recurrence (n = 84) and non-recurrence (n = 532). The baseline characteristics are summarized in Table 1. The cohort predominantly comprised males (58.6%), individuals aged < 60 years (58.3%), with a BMI < 25 kg/m^2^ (63.0%). Most patients reported no alcohol consumption (62.3%) or smoking (69.5%). A significant proportion had a CEAP grade ≥ 4 (58.3%) and no history of IVCS (61.7%). Few patients had a prior PC (6.7%), but a majority had a PCSD ≥ 3 months (83.0%). No significant differences were observed between the groups in terms of gender, alcohol consumption, smoking, hypertension, diabetes, IVCS, and PC (*P* > 0.05). However, age, BMI, CEAP grade, and PCSD differed significantly (*P* < 0.05) (Table 1).

**Table 1:** Patients’ characteristics.

Variables	Total	Recurrence	Non-recurrence	*P*
	(n = 616)	(n = 84)	(n = 532)	Re versus Non-re
Gender				0.238
Female	255 (41.7%)	40 (47.6%)	217 (40.8%)	
Male	361 (58.6%)	44 (52.4%)	315 (59.2%)	
Age (yrs)_				0.009
< 60	359 (58.3%)	38 (45.2%)	321 (60.3%)	
≥ 60	257 (41.7%)	46 (54.8%)	211 (39.7%)	
BMI (kg/m^2^)				0.002
< 25	388 (63.0%)	40 (47.6%)	348 (65.4%)	
≥ 25	228 (37.0%)	44 (52.4%)	184 (34.6%)	
Drinking				0.521
No	384 (62.3%)	36 (42.9%)	248 (46.6%)	
Yes	332 (37.7%)	48 (57.1%)	284 (53.4%)	
Smoking				0.677
No	428 (69.5%)	60 (9.7%)	368 (69.2%)	
Yes	188 (30.5%)	24 (90.3%)	164 (30.8%)	
Hypertension				0.116
No	474 (76.9%)	59 (9.6%)	415 (78.0%)	
Yes	14 (23.1%)	25 (90.4%)	117 (22.0%)	
Diabetes				0.076
No	577 (93.6%)	75 (87.8%)	502 (94.4%)	
Yes	39 (6.4%)	9 (12.2%)	30 (5.6%)	
CEAP grade				0.004
< 4	257 (41.7%)	47 (56.0%)	210 (39.5%)	
≥ 4	359 (58.3%)	37 (44.0%)	322 (60.5%)	
IVCS				0.135
No	380 (61.7%)	58 (69.4%)	322 (60.5%)	
Yes	236 (38.3%)	26 (30.6%)	210 (39.5%)	
PC				0.417
No	575 (93.3%)	77 (91.7%)	500 (94.0%)	
Yes	41 (6.7%)	7 (8.3%)	32 (6.0%)	
PCSD (mos)				0.016
< 3	105 (17.0%)	22 (26.2%)	83 (15.6%)	
≥ 3	511 (83.0%)	62 (73.8%)	449 (84.4%)	

Re recurrence, non-re non-recurrence, BMI body mass index, CEAP Clinical Etiological anatomical pathophysiological, IVCS Iliac Vein Compression Syndrome, PC Postoperative complication, PCSD Postoperative compression stocking duration.

### Establishment and validation of the nomogram model

**Figure 2:**
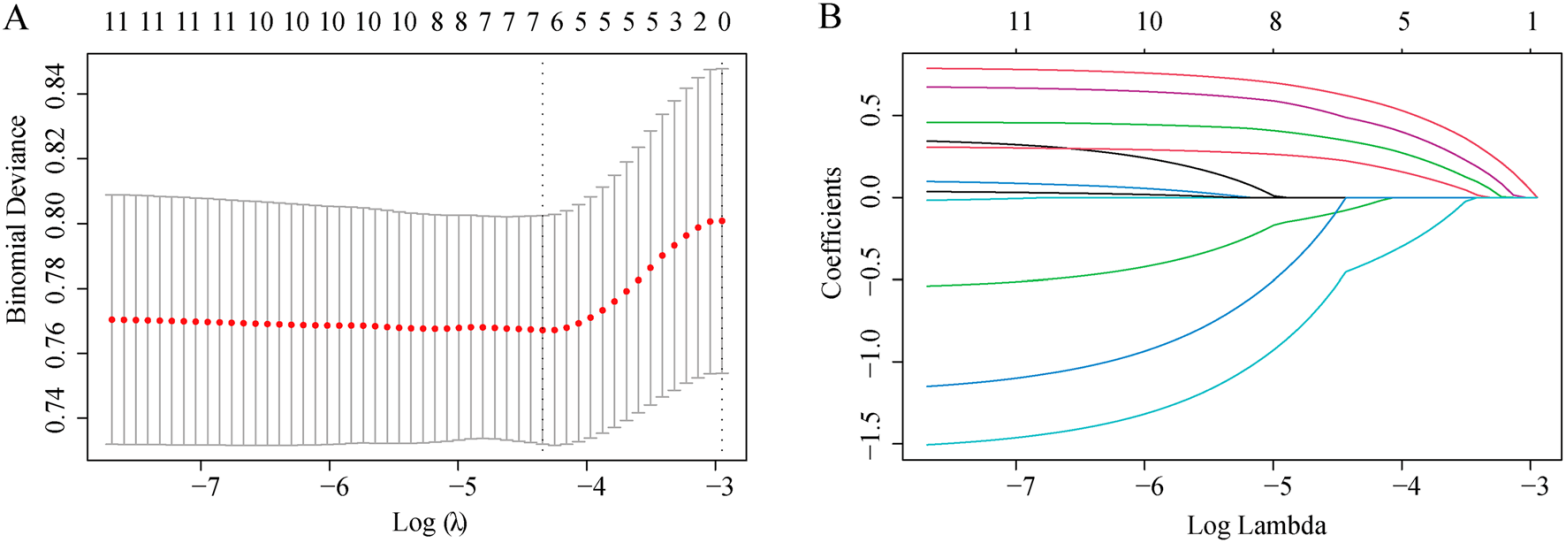
Variable selection by the LASSO cox logistic regression model. (**A**) 1000 bootstrap replicates by lasso Cox regression analysis for variable selection. (**B**) LASSO coefficients of clinical characteristics. Each curve represents a clinical characteristic.

**Figure 3:**
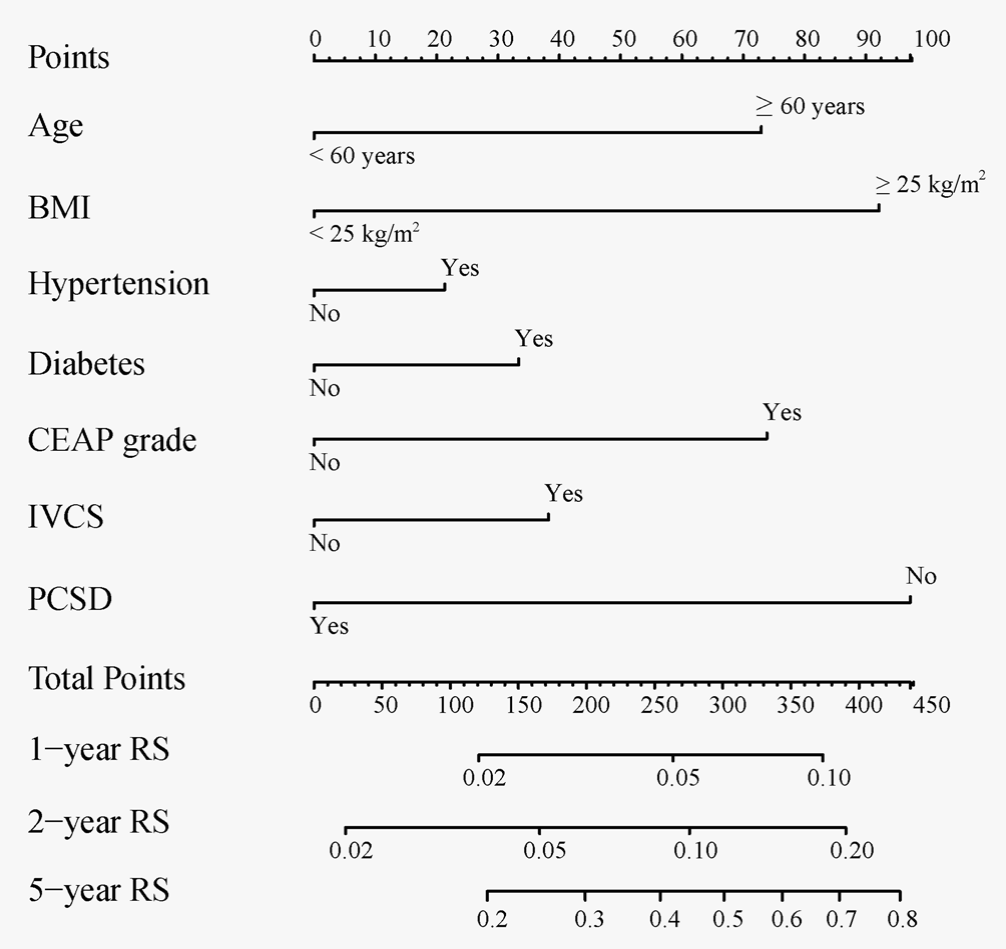
The graph showed nomogram for predicting 1-, 2-, and 5-year RS. BMI body mass index, IVCS Iliac Vein Compression Syndrome, PCSD Postoperative compression stocking duration, RS recurrence survival.

**Figure 4:**
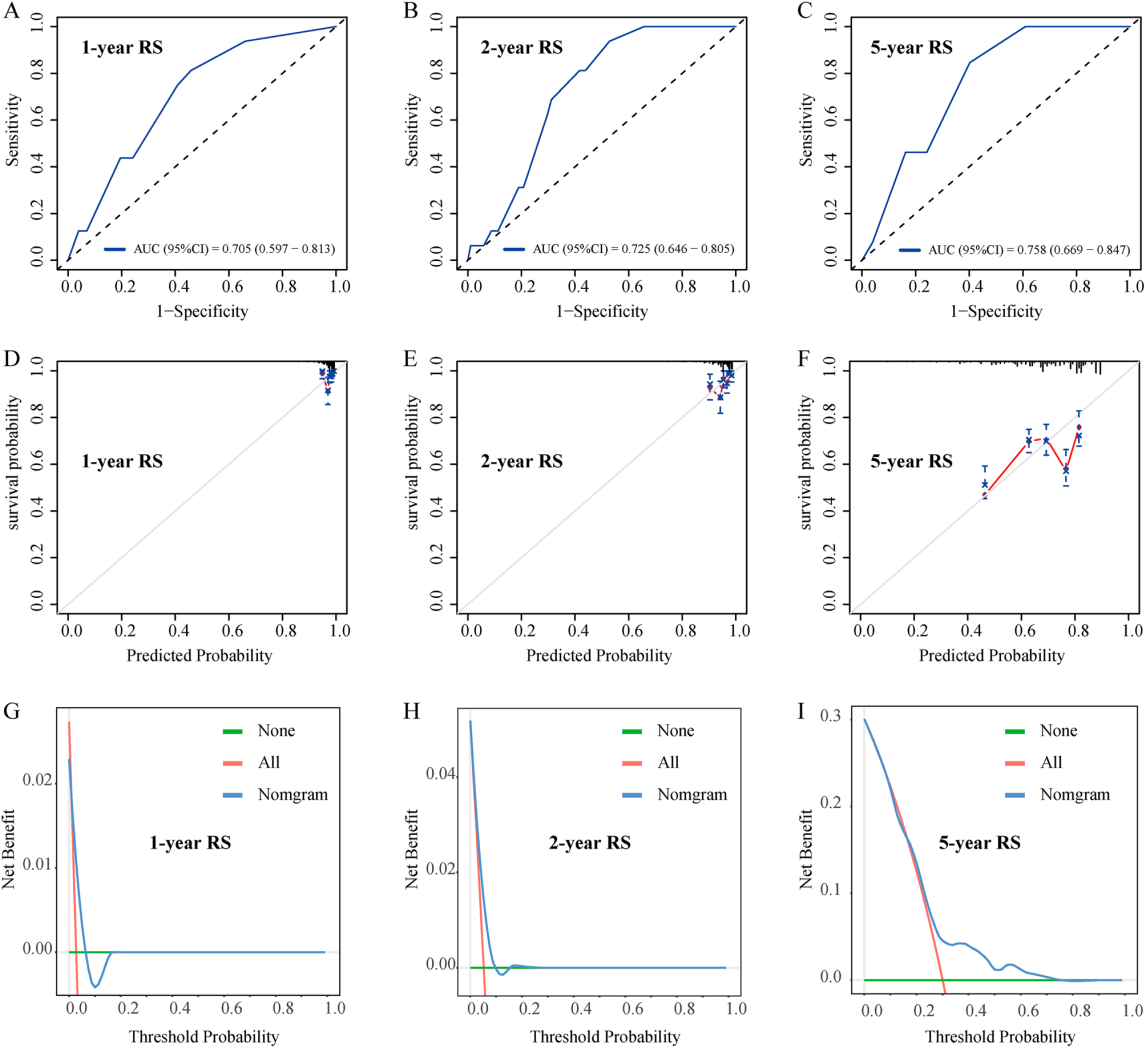
Model validation of ROC curve (**A**, **B**, and **C**), calibration plot (**D**, **E**, and **F**), and DCA (**G**, **H**, and **I**) for the 1-year, 2-year, and 5-year RS. ROC receiver operating characteristic curve, DCA decision curve analysis, RS, recurrence survival.

LASSO regression was employed to identify predictors from baseline characteristics (Fig. 2), reducing 11 variables to seven significant predictors: age, BMI, hypertension, diabetes, CEAP grade, IVCS, and PCSD. The LASSO coefficients of these variables are detailed in Table 2. A nomogram was developed to predict 1-, 2-, and 5-year RS post-VVLEs surgery (Fig. 3). The nomogram model allocated scores as follows: Age < 60 years (0 points), ≥ 60 (72.5 points); BMI < 25 kg/m^2^ (0 points), ≥ 25 kg/m^2^ (92.5 points); hypertension: no (0 points), yes (20 points); diabetes: no (0 points), yes (35 points); CEAP grade: no (0 points), yes (72.5 points); IVCS: no (0 points), yes (40 points); PCSD: yes (0 points), no (100 points). The total score, representing the sum of the points from these seven items, correlates with the individual risk of developing 1-, 2-, and 5-year RS post-VVLEs surgery. The model demonstrated adequate discriminative ability with a C-index of 0.716 (95%CI: 0.698–0.813). AUC values for 1-, 2-, and 5-year predictions were 0.705, 0.725, and 0.758, respectively, indicating good model performance (Fig. 4A–C). Calibration curves showed good agreement between predicted and observed outcomes (Fig. 4D–F). Decision Curve Analysis (DCA) indicated that the nomogram provided greater net benefits than standard care across various threshold probabilities (Fig. 4G–I).

**Table 2:** Selected Variables and Their Lasso Coefficients.

Variable	Lasso Coefficient
Age	0.303479314998822
BMI	0.466230682818106
hypertension	0.0620216199993233
diabetes	0.0948694924825136
CEAP grade	0.365652081766411
IVCS	0.167110569883559
PCSD	− 0.462923291869389

Selected variables and their lasso coefficients from cox proportional hazards model.

### Nomogram to stratify patient’s recurrence risk

**Figure 5:**
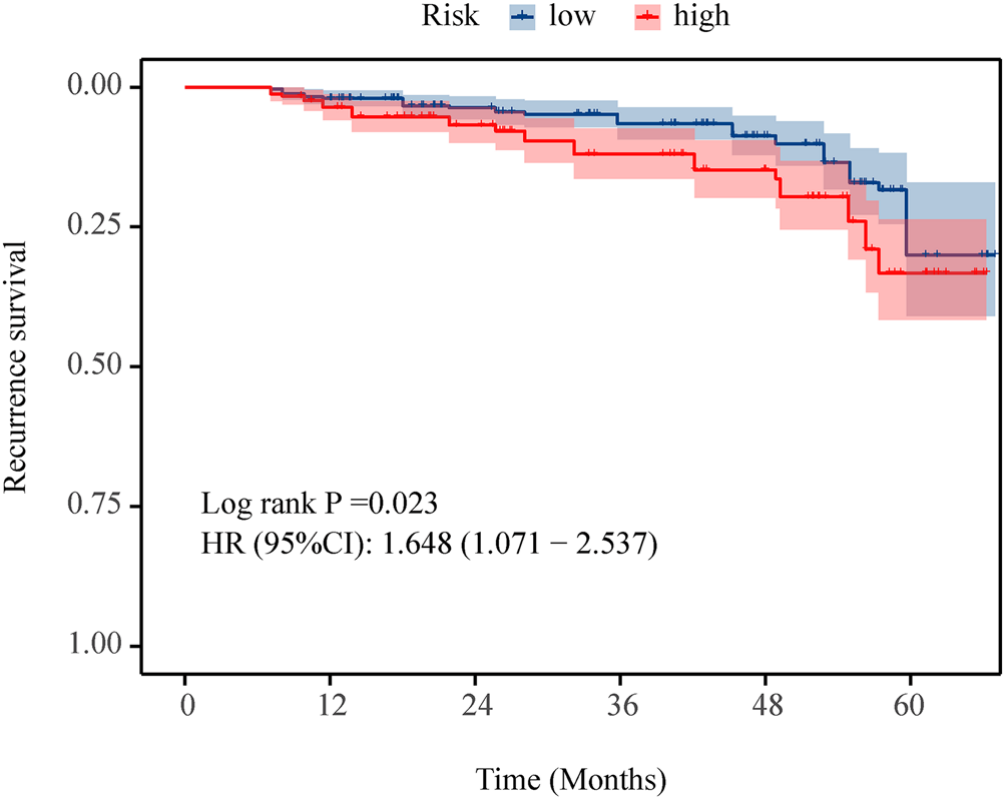
The Overall RS curves of the low-risk and high-risk groups in the entire Cohort. RS Recurrence survival.

Using a nomogram, we calculated the total score for each patient, which served as a basis for risk stratification. The optimal cutoff value for categorizing patients into different risk groups was established at 225, determined by the X-tile software analysis. Consequently, patients were divided into two main categories based on their total scores: those with scores of 225 or less were placed into the low risk subgroup, whereas patients with scores exceeding 225 were classified into the high risk subgroup. This stratification was further refined as patients were grouped into low-score and high-score categories according to the nomogram’s criteria. The impact of these risk categories on patient outcomes was evaluated using Kaplan–Meier analysis, coupled with a log-rank test, which revealed significant differences in the survival outcomes between the low and high-risk groups (*P* < 0.05), as illustrated in Fig. 5. Notably, patients within the high-risk group exhibited a significantly poorer prognosis when compared to their counterparts in the low-risk group. This clear demarcation between the groups underscores the utility of the nomogram.

## Discussion

This study enhances the existing body of knowledge by developing and validating a nomogram designed to predict the risk of recurrence following surgical procedures for VVLEs. Given the significant impact of VVLE recurrence on patient quality of life and healthcare costs, a reliable predictive tool is crucial for improving postoperative management and facilitating tailored treatment strategies.

In our analysis, we employed LASSO regression to identify key predictors of recurrence from a wide range of variables. We determined that age, BMI, hypertension, diabetes, CEAP grade, and IVCS are significant factors influencing the likelihood of recurrence, highlighting the multifaceted nature of VVLEs’ recurrence. Age, an unmodifiable factor, is associated with reduced venous wall elasticity and valvular dysfunction, aligning with increased postoperative recurrence risk reported in literature^21^. An increased BMI is linked to heightened venous system pressure, possibly leading to the formation and recurrence of varicosities^22^. Hypertension and diabetes, prevalent chronic conditions worldwide, may increase the risk of VVLEs recurrence by affecting the vascular wall and microcirculation^23,24^. Evidence suggests that these systemic diseases are linked to venous thrombosis through endothelial dysfunction and heightened inflammatory states, potentially leading to valvular incompetence^25–27^. The CEAP grade, which gauges the severity of VVLEs, demonstrates the impact of disease extent on recurrence risk^28^. IVCS, a common cause of lower extremity venous reflux, significantly increases the risk of postoperative recurrence by raising venous pressure and impeding venous return, potentially leading to new venous abnormalities and exacerbating symptoms of venous insufficiency^29^.

Our findings also highlight the duration of PCSD as an important predictor of recurrence, in agreement with previous studies^30–32^. Long-term use of compression stockings can reduce the risk of varicose vein recurrence by alleviating postoperative swelling, supporting the venous system, and minimizing blood stasis, thereby maintaining valvular function^33^. However, there is no consensus on the optimal duration of PCSD, suggesting that it should be tailored based on individual patient circumstances and risk factors, a customization our nomogram model supports.

By integrating these variables into the nomogram model, we provide an intuitive tool to calculate the probability of recurrence at various time points post-surgery for VVLEs patients. The model’s C-index of 0.716 indicates good predictive ability. The area under the ROC curve (AUC) demonstrates the model’s predictive capacity at 1, 2, and 5 years as 0.705, 0.725, and 0.758, respectively, signifying its accuracy in distinguishing between high and low-risk patients. The calibration curve’s high concordance further emphasizes the reliability of our predictions. Additionally, decision curve analysis (DCA) indicates that our nomogram has practical value in clinical decision-making, offering personalized treatment recommendations and preventing unnecessary or excessive treatment.

However, our study has limitations. Firstly, being retrospective, it may be subject to selection and information bias. Secondly, despite its good predictive performance, the model’s small sample size of recurrence cases, only 84, necessitates validation in larger-scale, multicenter studies. Furthermore, the diversity of surgical techniques prevented their consideration as a unified predictive factor, another limitation. Lastly, we acknowledge the need for future studies to delve into potential influencing factors, such as genetics, lifestyle, and socioeconomic aspects, to gain a more comprehensive understanding of the complex causes behind the recurrence of VVLEs post-surgery. It is also worth noting that integrating computational biology into VVLEs research could significantly enhance the understanding and management of the disease^11,34^. By delving into the roles of miRNAs and lncRNAs in gene regulation relevant to VVLEs, researchers have the potential to uncover genetic markers and new targets for treatment, paving the way for personalized medical approaches and improved treatment effectiveness^35–37^. Additionally, concentrating on the interactions between miRNAs and lncRNAs emerges as a promising avenue for advancing VVLEs management and patient care^38–40^. The employment of theoretical models based on ordinary differential equations (ODE) is particularly crucial in shedding light on the regulatory mechanisms within gene/protein signaling networks associated with VVLEs recurrence^41–43^. Such models offer profound insights into the complex biological systems governing variceal recurrence, highlighting the importance of these mechanisms for a deeper understanding of the disease’s causes^35,44,45^. By investigating these regulatory mechanisms, researchers are poised to identify novel therapeutic targets and intervention strategies, ultimately contributing to a more robust scientific foundation for VVLEs management. This approach is not only pivotal for enhancing our comprehension of the disease but also provides a crucial direction for future research aimed at predicting and managing VVLEs, thereby underscoring the significance of incorporating theoretical models into VVLEs research.

Despite these limitations, our study provides new insights into the postoperative management of VVLEs. Using our nomogram model, physicians can offer more accurate risk assessments and treatment recommendations to patients. Future research should focus on refining and validating the model and its applicability across different populations for broader clinical use.

In conclusion, the developed nomogram is a valuable tool for predicting postoperative recurrence risk in patients with VVLEs. By analyzing key predictive factors comprehensively, this model aids in guiding personalized patient management and treatment decisions. Reducing postoperative recurrence can enhance patients’ quality of life and decrease the burden on healthcare systems. We hope this model will be widely applied and enhanced in diverse clinical settings.

## Conclusion

This study successfully developed and validated a nomogram for predicting RS in patients with VVLEs after surgery. Integrating multiple important predictive factors, including age, BMI, hypertension, diabetes, CEAP classification, IVCS, and PCSD, this model holds significant clinical value. It enables physicians to provide more precise risk assessments and treatment recommendations for their patients.

## Data Availability

The primary data can be acquired from the corresponding authors in compliance with privacy and ethical constraints.
